# From Images to Words: How Packaging Style Affects Brand Preference in Heritage Food

**DOI:** 10.3390/foods14223858

**Published:** 2025-11-11

**Authors:** Haiyan Wang, Lingrong Lin, Honghai Wang, Xiaoye Jin, Chenhan Ruan

**Affiliations:** School of Economics and Management, Fujian Agriculture and Forestry University, Fuzhou 350007, China; gefu9@163.com (H.W.); lingrolin@163.com (L.L.); haigea@outlook.com (H.W.); 17504539044@163.com (X.J.)

**Keywords:** heritage food, representation style, food packaging, brand preference

## Abstract

Food, specifically those with heritage attributes, stands as one of the distinctive forms of Intangible Cultural Heritage (ICH). To promote and preserve such heritage, brands have increasingly focused on incorporating heritage elements into the packaging. This research employs three studies conducted in China to explore how different representation styles of heritage elements (verbal vs. non-verbal) shape consumer brand preferences in food packaging. Study 1 confirmed that food packaging featuring heritage elements effectively enhances consumer brand preference. Moreover, consumers exhibit stronger preference for the verbal elements over the non-verbal ones for heritage food due to construal level theory. Study 1 also demonstrated the mediating role of perceived value. Study 2 validated that such an effect remained significant within a tourism shopping context. In addition, Study 3 revealed the moderating effect of purchase motivation. When purchasing food as a gift, consumers tend to adopt a more abstract processing level (e.g., symbolic meaning, cultural connotation), which enhances the effect of verbal heritage elements on brand preference, whereas for self-use purchases, consumers shift to a concrete processing level (e.g., taste or price), thus enhancing the effect of non-verbal representation style. This research enriches the research on heritage element application in food marketing, and offers suggestions for packaging design for heritage food.

## 1. Introduction

The Convention for the Safeguarding of the Intangible Cultural Heritage (CSICH) defines “Intangible Cultural Heritage (ICH)” as various social practices, expressions, representations, knowledge, skills, as well as related tools, objects, crafts, and cultural spaces that are recognized by communities, groups, and sometimes individuals as part of their cultural heritage [1]. Food serves as a bridge connecting ICH with a broad market, providing a means for people to understand ethnic cultures [2] and evoking connections to their social and cultural identity [3]. Heritage food refers to traditional local foods that are rooted in various cultures, religions, and beliefs, and are inherited, prepared, and practiced on a daily basis [4]. Unlike products from other sectors, heritage food is characterized by its regional nature, forming cultural cues that help preserve cultural memory [5]. This makes heritage foods, like other heritage products, frequently developed as tourism souvenirs [6], marketed in a unique way centered around the culture of their place of origin. However, from a cultural perspective, intangible heritage is viewed as a shared cultural asset [7] that can be productized in regions outside its place of origin [8]. While this marketization approach helps promote intangible heritage culture, it also intensifies competition among intangible heritage products within the same category. To distinguish themselves from competitors, brands must find appropriate marketing positioning strategies for intangible heritage products, and the food sector is no exception. In addition, heritage food faces the threat of authenticity [4], with consumers questioning the methods of food preparation. Therefore, how to quickly communicate the value of heritage food to consumers and enhance product preference becomes an issue that needs to be addressed.

Packaging is a silent salesperson [9]. The elements on the package are the fundamental units that make up food packaging and have a significant impact on consumer choice [10]. Heritage food packaging, elements that highlight the cultural value are often used, leveraging the rarity of traditional craftsmanship as an effective marketing tool to enhance brand promotion [11]. According to research on packaging, packaging elements are primarily classified into verbal and non-verbal categories [12]. The former refers to the textual elements on the packaging, which are often associated with rational thinking processes. These elements can activate consumers’ heuristic reasoning and change their cognitive orientation [13]. In contrast, the latter refers to the graphic elements on the packaging, which can effectively trigger emotional responses in consumers [14,15,16], with a more significant impact during the emotional seeking phase [12]. Consumers often face time pressure while shopping [13], so brands need to understand consumers’ processing efficiency for different types of heritage packaging elements in order to design targeted packaging. Previous studies have shown that images tend to elicit stronger product evaluations from consumers compared to text [17], and non-verbal elements are more likely to trigger consumers’ purchase intentions [18]. However, based on construal level theory, the way individuals represent events depends on the psychological distance to the event [19]. This means that in the context of heritage food, consumers are more likely to adopt high-level construals when faced with heritage elements on packaging that are temporally distant. This is closer to textual information (which, when read, creates a greater psychological distance between the consumer and the described object) [20], leading to different results compared to previous studies. However, current research on heritage food mostly focuses on conceptual dimensions [4,21] and functional roles [22,23], with limited studies on the impact of different style elements in packaging. Therefore, this research, based on construal level theory, explores the impact of different heritage element representation styles (verbal vs. non-verbal) on consumer brand preferences.

At the same time, considering that verbal packaging elements can influence consumers’ perceived value of the product by conveying verbal cues [24], consumers are more likely to engage in fluent processing under the same construal level, making them more receptive to the cultural cues conveyed by food packaging elements. Additionally, people often purchase heritage products as gifts when traveling. Therefore, in this research, perceived value is treated as a mediating variable, with purchase motivation as a moderating variable, to explore their roles in the marketing process of heritage food. We reviewed the literature on food packaging, heritage element representation style, perceived value, and purchase motivation, and based on this, we proposed four hypotheses: main effects, mediating effects, and moderating effects. These hypotheses were tested through three situational studies. We selected real-life heritage food products as stimuli for Studies 1 and 2, including Wuyi Rock Tea, whose tea-making technique is listed on UNESCO’s Representative List of the Intangible Cultural Heritage of Humanity, and Gulangyu Pie, whose production technique is included in the seventh batch of the Provincial Intangible Cultural Heritage Representative List of Fujian Province. To eliminate the potential confounding effect of product familiarity, we also created a virtual rice brand based on traditional production techniques as the stimulus for Study 3. Finally, we discussed the research conclusions, managerial implications, and future research directions. This research provides a new perspective for the packaging design of heritage food and contributes to the promotion and innovative development of intangible heritage culture.

## 2. Related Literature and Hypotheses Development

### 2.1. Food Packaging and Heritage Element Representation Style

Packaging is a critical component in the food industry [25]. Food packaging carries marketing attributes, informing consumers about the product’s characteristics and offering more personalized services [26]. From discussing the role of packaging [27] to exploring its influence on consumers [28], current research on food packaging primarily integrates multiple fields, including marketing, logistics supply chain management, food technology, and the environment [26]. For example, research focuses on the impact of packaging on supply chain transportation efficiency and environmental protection [29], as well as the role of sustainable packaging in reducing food waste and preventing chemical contamination [30]. Like products in other sectors, the images, colors, weight, and even the sound of packaging can influence consumers’ perceptions, ultimately affecting the overall consumption experience [31]. Food packaging also contains both verbal and non-verbal elements. Verbal elements effectively convey information to consumers, while non-verbal elements shape expectations about the product’s attributes. Consumers may consciously or unconsciously use images to infer product information [32]. As a result, images are often used on food packaging to attract consumer attention and enhance purchase intentions [33]. Meanwhile, research conducted after extensive surveys emphasizes that informational elements on food packaging are more important than visual elements [25]. In summary, the impact of verbal and non-verbal elements on consumers in food packaging may vary.

In previous studies, intangible heritage elements primarily referred to the unique cultural heritage value of tourism destinations [34], and were used in research on the competitiveness of these destinations [35,36]. In this research, since the research context is food packaging, heritage elements are combined with packaging elements, and heritage elements are defined as those elements on food packaging that convey the unique cultural heritage value of the food. The heritage element representation style refers to the stylistic form of the heritage elements on the packaging. According to the CSICH, intangible cultural heritage is passed down through generations, continuously recreated within communities and groups as they adapt to their surroundings and interact with nature and history. This process provides these communities and groups with a sense of identity and continuity, thereby enhancing respect for cultural diversity and human creativity [1]. Due to the regional characteristics of intangible heritage culture, its use in packaging is often seen in tourism souvenirs [5], and related research primarily focuses on commercialization, intellectual property, and other topics [37,38]. From a marketing perspective, intangible heritage labels and narrative expressions have been shown to enhance consumers’ perceived value of products [11,39], thus confirming the cultural role of intangible heritage in marketing. Heritage elements on food packaging can serve as carriers for the transmission of intangible heritage, highlighting the cultural value and historical depth of the food. The content carried by these elements is the crystallization of wisdom accumulated over time, and for consumers, these elements are distant in time and history. Therefore, a straightforward and simple presentation is needed to reveal their inherent value. Both text and graphics are crucial components of visual communication [40].

### 2.2. The Influence of Heritage Element Representation Style on Consumer Brand Preference

Heritage food is a material manifestation of culture, showcasing the diversity of human “living heritage” [41]. For consumers within a region, their love and pride for their country often lead them to support and prefer domestic brand products [42]. For consumers outside the region, the local culture embedded in heritage food reflects the uniqueness of its place of origin. As a carrier of shared cultural significance, it can enhance consumers’ behavioral intentions by fostering a broader sense of collective identity [7]. This cultural connection enables individuals to form a self-product identity link, leading to positive evaluations and emotional responses toward the product [43]. In marketing applications, when consumers encounter heritage elements on food packaging, it is likely to activate a cultural identity connection between the individual and intangible culture, leading to an attitude that supports this social identity. That is, food packaging featuring heritage elements is more likely to elicit consumer preference than packaging without such elements. This forms the first hypothesis of this study.

**H1.**  *Food packaging with heritage element increases consumer brand preference than packaging without heritage element*.

According to construal level theory, individuals’ mental representations take two forms: high-level construal, which is decontextualized, core, and abstract, and low-level construal, which is contextualized, surface, and concrete [44]. There is a connection between psychological distance and construal level. Time distance, spatial distance, social distance, and probability all fall under the category of psychological distance, relying on an individual’s direct experience [45]. For events with greater psychological distance (e.g., those that involve more distant subjects), individuals tend to adopt high-level construals. In contrast, for events with closer psychological distance, individuals are more likely to adopt low-level construals [45].

In real-world contexts, images and text are closely linked to different construal levels. Images have a high degree of physical similarity to the objects they represent, and people tend to analyze image information by perceiving the real object, meaning the process of analyzing image information is similar to that of analyzing the actual object, thus conveying a sense of closer psychological distance [46]. In contrast, text conveys information that remains consistent regardless of time or location, and reading text tends to create a sense of greater psychological distance [47]. Text information captures the core features of an object, while images preserve the specific details of an event, reflecting the mental representations of high-level and low-level construals, respectively.

Research indicates that when words representing psychological distance are paired with words reflecting construal level in a consistent manner, participants’ response times are significantly shorter compared to when the pairings are inconsistent [48]. From the perspective of time distance, intangible heritage culture, with its origins in distant history, relates to events that occur in the distant future. Textual elements associated with high-level construals are more likely to influence consumer attitudes. Additionally, some scholars have introduced the concept of information distance, suggesting that the more product information consumers have, the smaller the information distance, leading them to evaluate products based on low-level construals [49]. Since consumers are often unaware of the production techniques behind heritage food, they experience a larger information distance and require high-level construals to understand the event. This leads to the formulation of the second hypothesis.

**H2.**  *Food packaging with verbal (vs. non-verbal) heritage elements leads to stronger consumer brand preference.*

### 2.3. The Mediating Effect of Perceived Value

Perceived value is the post-consumption perception of outcome, referring to the perception of consumption value or use value [50]. Most scholars measure perceived value through multiple dimensions, and different levels of perceived value have varying impacts on different categories of products. When consuming cultural products, consumers tend to focus more on emotional and symbolic value than on utilitarian value [50]. According to the expectancy theory of motivation, consumers are more likely to make positive consumption decisions when they expect engaging in a specific cultural consumption to yield attractive outcomes. In this research, heritage food is considered a cultural product that expresses, distinguishes, and symbolizes personal identity [51], facilitating the communication of a positive image to others in society and obtaining social value. The perceived social value derived from cultural products is a major determinant of consumers’ purchase intentions [52] and can significantly enhance product utility evaluations [53]. Perceived social value has a notable impact on consumers’ willingness to engage with food brands [54]. Verbal elements that convey intangible heritage culture on food packaging, due to their direct representation style, allow readers to clearly understand the cultural information being communicated. The intangible heritage cultural content they convey is embedded with the emotional and societal perspectives of preserving traditional cultures, which easily enhances consumers’ perceived cultural value when purchasing related products. Moreover, purchasing products with packaging featuring heritage elements can highlight the positive aspects of cultural preservation, symbolizing an individual’s cultural protection awareness and showcasing societal value.

Non-verbal information can provide consumers with a positive aesthetic experience through visual communication design, maximizing their aesthetic value and expanding their perceived value, thereby triggering their desire to purchase. In the context of heritage food, unique and precise graphic design helps consumers appreciate the beauty of intangible heritage culture. However, poorly executed graphic content can be influenced by consumers’ subjective interpretations, leading to biases and product avoidance, which may result in lower perceived value of the product. In summary, non-verbal representation of intangible heritage culture contain inherent instability, whereas verbal intangible heritage packaging information tends to convey higher perceived value, leading to the formulation of the third hypothesis.

**H3.**  *Perceived value mediates the relationship between heritage element representation style and consumer brand preference.*

### 2.4. The Moderating Effect of Purchase Motivation

People’s motivation in purchasing activities are often divided into personal use or gift-giving, and different purchase motivation directly affect consumers’ information processing modes, purchase involvement, imagination of product use, and purchase decisions [55]. From the perspective of construal level theory, consumers who purchase for personal use tend to have a lower level of construal, while those who purchase as gift-giving tend to have a higher level of construal [56]. Under the purpose of gift-giving, consumers often tend to adopt an elaboration processing mode [57] and choose popular products to reduce consumption risk [58], with a primary focus on external cues such as product packaging. Consumers consider how much potential utility the recipient will derive from each gift [59], emphasizing abstract meanings such as the symbolic, cultural, and commemorative significance conveyed by the gift. Therefore, decontextualized verbal elements are more consistent with consumers’ preferences in gift-giving contexts. In contrast, when purchasing for personal use, consumers are driven by utilitarian motivations and pay more attention to the concrete attributes of the product. Thus, concrete non-verbal elements, such as images, are more appropriate for products intended for personal consumption.

Based on construal level theory, when words representing construal levels are consistent with those representing psychological distance, consumers’ response times are significantly shorter. When purchasing for gift-giving, consumers exhibit a higher construal level, which corresponds to the distant psychological cues conveyed by verbal elements. In contrast, when purchasing for personal use, consumers exhibit a lower construal level, which corresponds to the proximal psychological cues conveyed by non-verbal visual elements. Therefore, the fourth hypothesis is proposed.

**H4.**  *Purchase motivation moderates the relationship between the heritage element representation style of food packaging and consumer brand preference. When the purchase motivation is gift-giving, the verbal heritage element representation style evokes a stronger consumer brand preference among consumers. Conversely, when the purchase motivation is personal use, the non-verbal heritage element representation style elicits a stronger consumer brand preference*.

**Figure 1:**
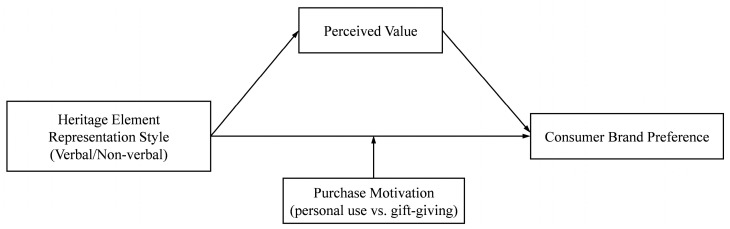
Conceptual model.

Based on the above hypotheses, a theoretical model is constructed (as shown in Figure 1).

## 3. Materials and Methods

### 3.1. Overview of Experimental Designs

We tested our hypotheses through three studies. Study 1 was conducted with 180 participants, aiming to test our basic proposition that compared with food packaging without heritage elements, packaging containing heritage elements elicits higher consumer brand preference (H1). Furthermore, packaging that adopts a verbal (vs. non-verbal) heritage element representation style exerts a more positive effect on consumer brand preference (H2). It also verified the mediating role of perceived value in the main effect (H3), while ruling out alternative explanations related to packaging attractiveness, informativeness, cultural authenticity, processing fluency, and emotional arousal. Study 2 recruited 150 participants, with the goal of testing whether the main and mediating effects found in Study 1 still held in the context of purchasing souvenirs for tourism. Study 3 recruited 140 participants, aiming to demonstrate the moderating effect of purchase motivation (H4). In summary, these three studies provide consistent support for the conceptual framework we proposed.

### 3.2. Study 1

The goal of Study 1 was to explore the impact of intangible heritage representation styles in food packaging on consumer brand preference (H1 and H2), and verified the mediating role of perceived value (H3). Study 1 employed a one-factor three-level (verbal vs. non-verbal vs. control group) between-subjects design. Data was collected through the Credamo platform, with a total of 180 participants recruited. Of the participants, 61 were male (33.9%), 105 were aged 21–30 (58.3%), and 57 had a monthly consumption level ranging from 140 to 421 USD (31.7%). Prior to participation, each participant signed an online informed consent form.

#### 3.2.1. Method

**Figure A1:**
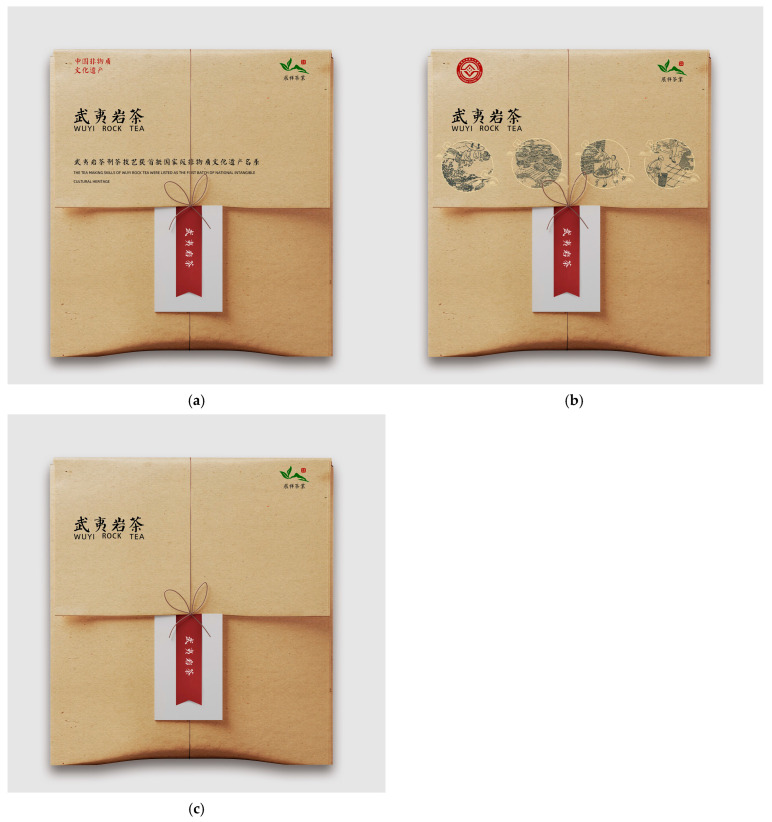
(**a**) Verbal group product packaging image (The top-left corner of the packaging is labeled “China Intangible Cultural Heritage,” and the packaging directly mentions “Production techniques listed as National Intangible Cultural Heritage Register.”); (**b**) Non-verbal group product packaging image (This packaging contained the symbol of Chinese intangible cultural heritage and illustrations of the tea-making process.); (**c**) Control group product packaging image (This packaging did not contain any heritage elements).

The experimental stimuli were packaging images of Wuyi Rock Tea (as shown in Figure A1), which uses intangible heritage tea-making techniques. Participants were informed that they would be involved in a study on new product packaging. The study was divided into three parts. In the first part, participants were randomly assigned to one of the following three groups: the verbal heritage elements group (in this group, the packaging directly mentioned “Production techniques listed as National Intangible Cultural Heritage Register”), the non-verbal heritage elements group (this group’s packaging contained the symbol of Chinese intangible cultural heritage and illustrations of the tea-making process), or the control group (this group’s packaging did not contain any heritage elements). Participants were then asked to view the product packaging for 15 seconds and imagine themselves in the following scenario: “Your household tea supply is running low, and you are planning to buy new tea. You and your family have no particular brand preference, nor are you strictly seeking a specific type of tea. At the same time, you and your family have sufficient funds for this tea purchase. While browsing online, you come across the following product.”

In the second part, participants were asked to rate the product packaging they viewed, including the assessment of the presence of verbal and non-verbal heritage elements, perceived attractiveness [60], perceived informativeness [61], and perceived cultural authenticity [62]. Participants then filled out a series of scales: consumer brand preference scale (α = 0.90), brand purchase intention scale (α = 0.88), perceived value scale (α = 0.94), processing fluency scale (α = 0.79), emotional arousal scale (α = 0.86) (the items of the scale are shown in Table 1). All variables were measured using a 7-point Likert scale. Finally, in the third part, participants reported their demographic information.

#### 3.2.2. Results

*Manipulation Check.* The results of the independent samples t-test indicate a significant difference in scores between the verbal and non-verbal heritage elements. In terms of language perception scores, the verbal group scored higher (M_verbal_ = 6.13, SD = 0.87; M_non-verbal_ = 3.53, SD = 1.58, t(118) = 11.17, *p* < 0.001). In terms of non-verbal perception, the non-verbal group scored higher (M_verbal_ = 2.58, SD = 1.39; M_non-verbal_ = 5.60, SD = 1.03, t(118) = −13.49, *p* < 0.001). These results indicate that the two types of heritage element style stimuli were successfully tested. In addition, the results of the participants’ ratings of the perceived attractiveness show no significant difference between the verbal and non-verbal groups (t(118) = −0.47, *p* = 0.64). The participants’ ratings of the perceived informativeness also showed no significant difference between the verbal and non-verbal groups (t(114) = 1.47, *p* = 0.15). The participants’ ratings of the packaging cultural authenticity also revealed no significant difference between the verbal and non-verbal groups (t(115.7) = 0.00, *p* = 1.00). These results eliminate alternative explanations based on attractiveness, informativeness, and cultural authenticity.

**Figure 2:**
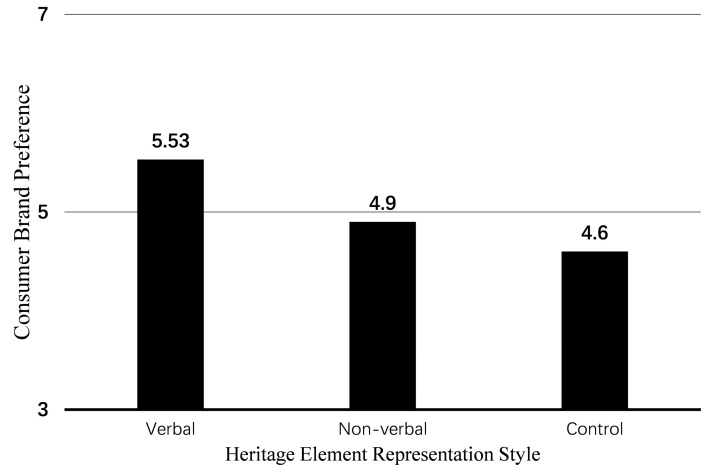
The impact of heritage element representation styles on consumer brand preference in Study 1.

*Main Effect.* Using the heritage element representation style as the independent variable and consumer brand preference as the dependent variable, the results of the one-way ANOVA show that the main effect of heritage elements is significant (F(2, 177) = 12.05, *p* < 0.001). The between-group comparison shows that participants in the verbal group (M_verbal_ = 5.53, SD = 0.88) have significantly higher brand preference scores than participants in the non-verbal group (M_non-verbal_ = 4.90, SD = 1.02; t(115.5) = 3.61, *p* < 0.001) and the control group (M_control_ = 4.60, SD = 1.26; t(105.5) = 4.72, *p* < 0.001). Participants in the non-verbal group scored higher than those in the control group, but the difference between these two groups was not significant (t(118) = 1.47, *p* = 0.14) (as shown in Figure 2). These results support H1 and H2, indicating that food packaging with heritage cultural representation elements is more likely to increase consumer brand preference compared to packaging without such elements, with the verbal heritage element representation style (vs. non-verbal) being more effective in enhancing consumer brand preference.

**Figure 3:**
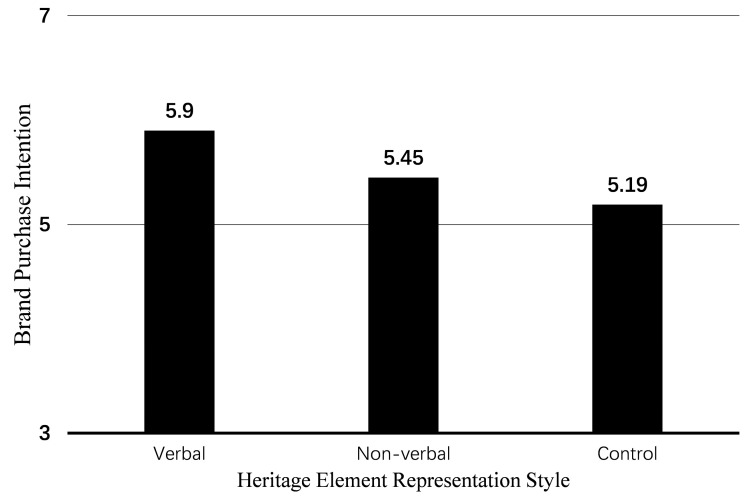
The impact of heritage element representation styles on brand purchase intention in Study 1.

Using the heritage element representation style as the independent variable and brand purchase intention as the dependent variable, the results of the one-way ANOVA show that the main effect of heritage elements is significant (F(2, 177) = 7.14, *p* = 0.001). The between-group comparison shows that participants in the verbal group (M_verbal_ = 5.90, SD = 0.83) have significantly higher brand purchase intention scores than participants in the control group (M_control_ = 5.19, SD = 1.29; t(100.8) = 3.56, *p* = 0.007) and the non-verbal group (M_non-verbal_ = 5.45, SD = 1.02; t(118) = 2.79, *p* = 0.006). Participants in the non-verbal group scored higher than those in the control group, but the difference between these two groups was not significant (t(118) = 1.25, *p* = 0.216) (as shown in Figure 3). These results suggest that verbal heritage elements can enhance consumers’ purchase intention for heritage products, while the presentation of non-verbal heritage elements does not trigger this effect, thus supporting H2 from a behavioral perspective.

**Figure 4:**
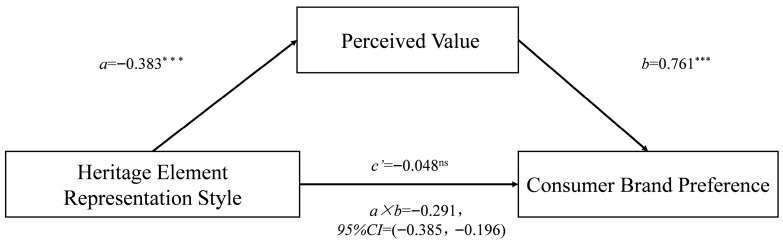
The mediating effect of perceived value in Study 1. (The asterisks following the path coefficients indicate significance levels: *** denotes *p* < 0.001, and ^ns^ denotes *p* > 0.05.)

*Mediation effect.* Using perceived value as the mediating variable, a mediation test was conducted following Hayes’ Bootstrap method (Process Model 4, sample size = 5000) [68]. The results show that perceived value significantly mediates the effect of heritage element representation style on consumer brand preference (β = −0.291, SE = 0.047, 95% CI = [−0.385, −0.196]), with a mediation effect size of −0.291 (as shown in Figure 4). After controlling for perceived value, the direct effect of heritage element representation style on consumer brand preference was not significant (β = −0.066, SE = 0.070, 95% CI = [−0.204, 0.071]), indicating that perceived value fully mediates the effect, thus validating H3.

**Figure 5:**
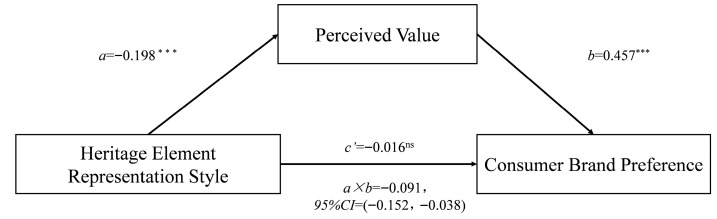
The mediating effect of perceived value after controlling for processing fluency and emotional arousal in Study 1. (The asterisks following the path coefficients indicate significance levels: *** denotes *p* < 0.001, and ^ns^ denotes *p* > 0.05.)

With emotional arousal and processing fluency as covariates, the mediating effect of perceived value remains significant (β = −0.091, SE = 0.029, 95% CI = [−0.152, −0.038]) (as shown in Figure 5). Therefore, the alternative explanatory roles of emotional arousal and processing fluency can be excluded.

#### 3.2.3. Discussion

Study 1 confirmed that the observed effect was driven by the heritage element representation style on food packaging by adding a control group and ruling out alternative explanations. Unlike previous studies suggesting that non-verbal (vs. verbal) elements tend to elicit more positive consumer responses [17,18], in the context of heritage food packaging, verbal heritage elements were found to enhance consumer brand preference to a greater extent, with perceived value serving as a mediating factor in this relationship. Although Study 1 demonstrated a certain brand preference for heritage food among consumers, tea products are generally priced higher, and heritage foods are often local specialties with characteristics of tourist souvenirs. Therefore, subsequent studies will further explore whether lower-priced heritage foods apply to the above main effects and mediation mechanism in a tourism context.

### 3.3. Study 2

Study 1 verified the effect of heritage element representation style on consumer brand preferences in a daily consumption context. However, as heritage foods are characterized by regional and cultural attributes, they are often developed as souvenirs. People are more likely to encounter heritage foods in tourism contexts. Therefore, Study 2 aimed to examine whether the main effect and mediating effect identified in Study 1 would remain robust in a tourism setting.

#### 3.3.1. Pretest

**Figure A2:**
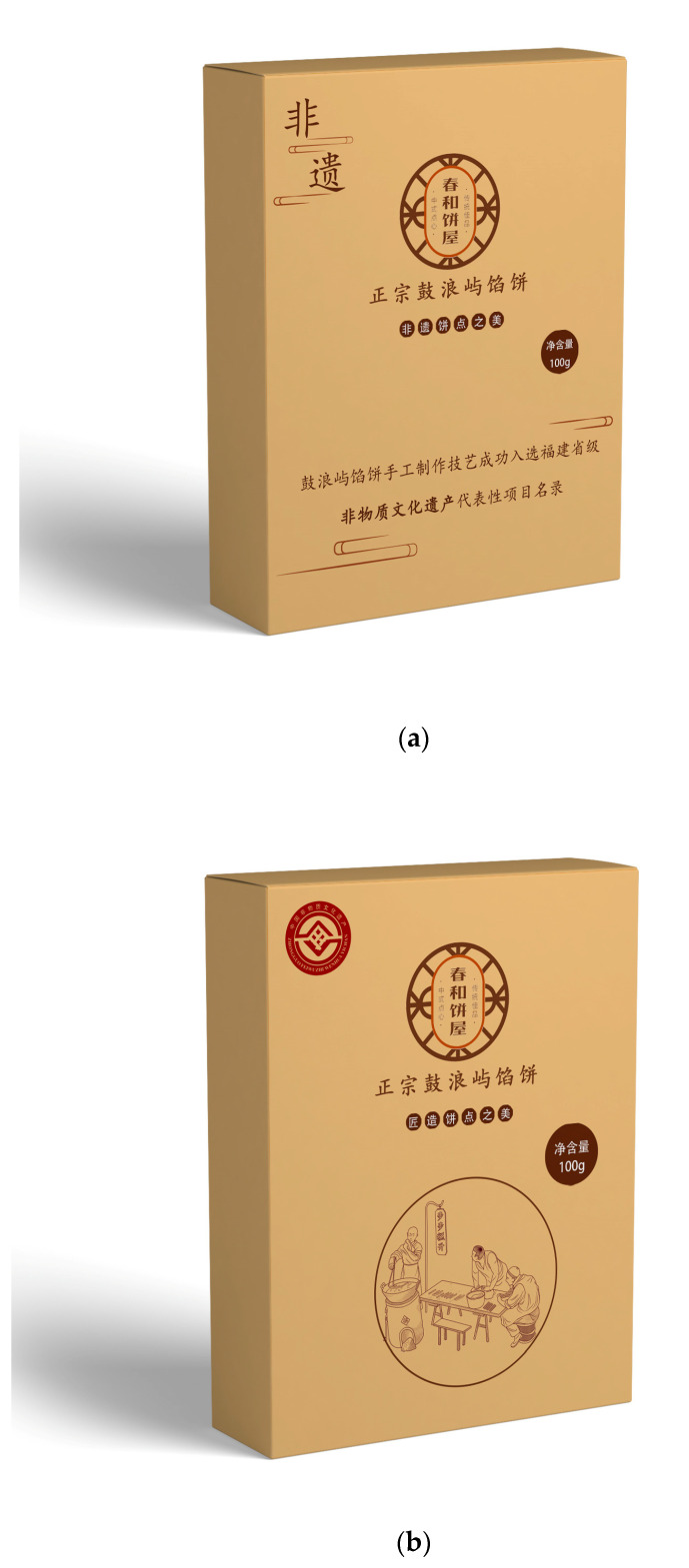
(**a**) Verbal group product packaging image(The top-left corner of the packaging is labeled “Intangible Cultural Heritage,” and at the bottom of the packaging it states: “the production technique successfully included in the Fujian Provincial Intangible Cultural Heritage Representative Project List”); (**b**) Non-verbal group product packaging image(The packaging featured the Chinese Intangible Cultural Heritage logo and illustrations of the production process).

The pretest aimed to select appropriate heritage food packaging images for Study 2. Since the results of Study 1 already demonstrated that food packaging with (vs. without) heritage element increases consumer brand preference, Study 2 no longer include a control group. Study 2 designed packaging featuring verbal and non-verbal heritage elements for Gulangyu Pie, a heritage food whose production technique has been included in the seventh batch of Fujian Provincial Intangible Cultural Heritage Representative Projects (as shown in Figure A2). A total of 50 participants were recruited from Credamo (16 males, 32%; 28 aged between 21 and 30, 56%; 15 with a monthly consumption level between USD 140–421, 30%) to take part in this pretest. Prior to participation, each participants signed an informed consent form online. The pretest adopted a single-factor, two-level (verbal vs. non-verbal) between-subjects design. The participants were randomly assigned to either the verbal heritage element group (where the packaging directly mentioned “the production technique successfully included in the Fujian Provincial Intangible Cultural Heritage Representative Project List”) or the non-verbal heritage element group (where the packaging featured the Chinese Intangible Cultural Heritage logo and illustrations of the production process). After viewing either the verbal or non-verbal heritage packaging images, all participants were asked to rate the packaging, specifically evaluating the determination of verbal/non-verbal heritage elements, perceived attractiveness, perceived informativeness, and perceived cultural authenticity.

The independent samples t-test results showed that there was a significant difference in the scores between verbal heritage elements and non-verbal heritage elements in the experimental materials of Study 2. In terms of verbal perception scores, the verbal group scored higher (M_verbal_ = 6.08, SD = 0.70; M_non-verbal_ = 4.28, SD = 1.31, t(36.8) = 6.06, *p* < 0.001). In terms of non-verbal perception scores, the non-verbal group scored higher (M_verbal_ = 3.68, SD = 1.60; M_non-verbal_ = 5.32, SD = 1.31, t(46.3) = −3.96, *p* < 0.001). This indicates that the two types of heritage element representation style stimuli passed the test. In addition, the results of participants’ ratings on packaging attractiveness showed no significant difference between the verbal and non-verbal groups (t(48) = −1.36, *p* = 0.18). The results of participants’ ratings on packaging informativeness also showed no significant difference between the verbal and non-verbal groups (t(48) = 0.12, *p* = 0.91). The results of participants’ ratings on packaging cultural authenticity showed no significant difference between the verbal and non-verbal groups (t(48) = −0.42, *p* = 0.68). Therefore, the participants were able to distinguish the heritage element representation styles used in the materials designed for Study 2, and the alternative explanations related to packaging attractiveness, informativeness, and cultural authenticity were ruled out.

#### 3.3.2. Formal Experimental Design

A single-factor between-subjects design was used, comparing verbal vs. non-verbal heritage elements. A total of 150 participants were recruited through Credamo, with 149 valid responses collected after excluding one participant who failed the screening question. Among the participants, 42 were male (28.2%), 91 were aged 21–30 years (61.1%), and 92 had a monthly consumption level between $701.5 and $982 (61.7%). Before participating, each participant signed an informed consent form online.

#### 3.3.3. Method

The experimental materials were consistent with those tested in the pretest, selecting the heritage food from the Gulangyu Scenic Area (a national 5A-level scenic area in Xiamen, Fujian Province, China)—Gulangyu pie. Participants were first asked to watch and read the scenario materials, imagining that they were purchasing souvenirs in the Gulangyu tourism area in Xiamen. They were then randomly assigned to either the verbal or non-verbal representation style packaging of the product. After viewing the product for 15 seconds, participants completed the brand preference scale, perceived value scale, and manipulation check items. Finally, participants provided their demographic information. The items related to perceived value, brand preference, and manipulation checks were consistent with those used in Study 1.

#### 3.3.4. Results

*Manipulation check.* In terms of verbal perception scores, the verbal group scored higher (M_verbal_ = 6.03, SD = 0.92; M_non-verbal_ = 4.75, SD = 1.37, t(130) = 6.71, *p* < 0.001); for non-verbal perception, the non-verbal group scored higher (M_verbal_ = 3.82, SD = 1.50; M _non-verbal_ = 5.16, SD = 1.43, t(147) = −5.55, *p* < 0.001). This indicates that the two types of heritage element representation style stimuli passed the test.

*Main effect test.* A one-way analysis of variance was conducted with consumer brand preference as the dependent variable. The results show that the consumer brand preference in the verbal group (M_verbal_ = 5.31, SD = 0.64) was higher than that in the non-verbal group (M_non-verbal_ = 4.94, SD = 0.95; F(1, 147) = 7.75, *p* = 0.006), further confirming the main effect of the study.

**Figure 6:**
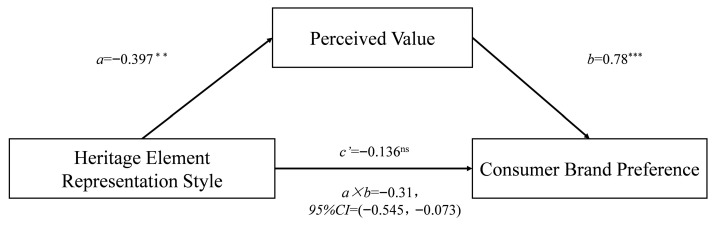
The mediating effect of perceived value in Study 2. (The asterisks following the path coefficients indicate significance levels: *** denotes *p* < 0.001, ** denotes *p* < 0.05, and ^ns^ denotes *p* > 0.05.)

*Mediation effect test.* The heritage element representation style was used as the independent variable, consumer brand preference as the dependent variable, and perceived value as the mediating variable. The mediation effect was tested using the Bootstrap method (Process Model 4, sample size = 5000). The results show that perceived value significantly mediated the effect of heritage element representation style on consumer brand preference (β = −0.31, SE = 0.12, 95% CI = [−0.545, −0.728]), with a mediation effect size of −0.31. After controlling for perceived value, the direct effect of heritage element representation style on consumer brand preference was not significant (β = −0.113, SE = 0.084, 95% CI = [−0.280, 0.054]) (as shown in Figure 6), confirming that perceived value fully mediates the effect, further verifying H3.

#### 3.3.5. Discussion

Whereas previous studies in tourism contexts have primarily focused on the contribution of heritage element diversity to the development of local tourism [36], Study 2 examined heritage elements in souvenir packaging and validated both the main and mediating effects through an online experiment. The findings demonstrate the practical applicability of the research effects in the promotion of heritage food products and highlight the role of intangible cultural heritage in supporting tourism development from the perspective of souvenir development. Study 3 will further explore the boundary conditions of these effects.

### 3.4. Study 3

Study 3 aims to verify whether the different purchase motivations of consumers when purchasing heritage food moderate the effect of heritage element representation style on consumer brand preference (H4).

#### 3.4.1. Pretest

**Figure A3:**
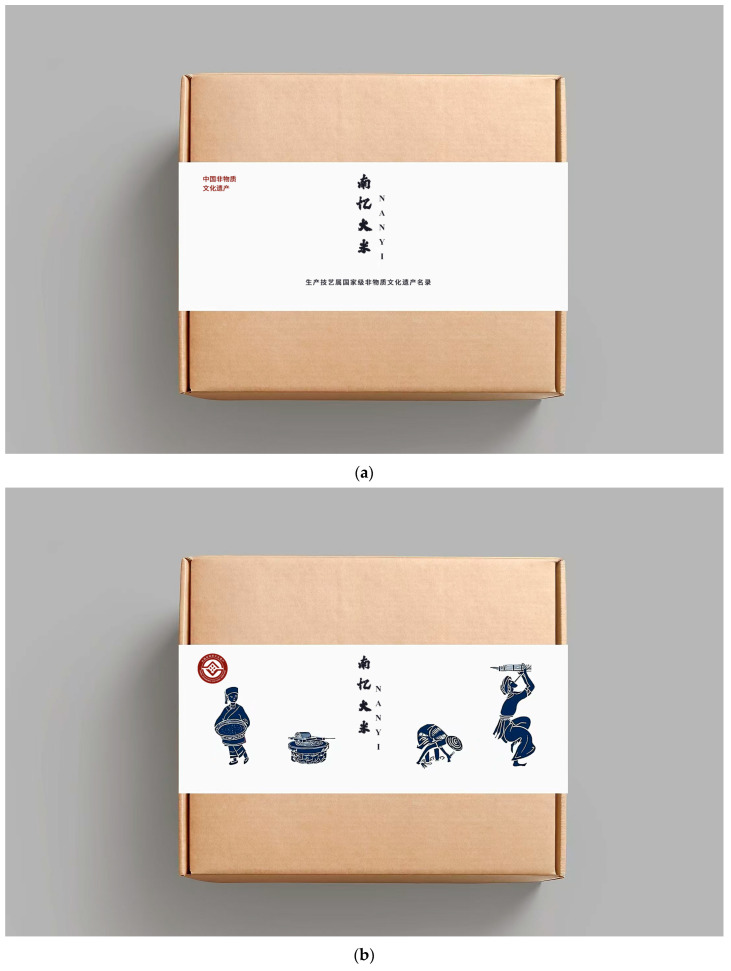
(**a**) Verbal group product packaging image (The top-left corner of the packaging indicates “China Intangible Cultural Heritage,” the product name “Nanyi Rice” is displayed in the center, and the bottom of the packaging states “Production techniques listed as National Intangible Cultural Heritage Register.”); (**b**) Non-verbal group product packaging image (This packaging contained the symbol of Chinese intangible cultural heritage and illustrations of the production process.).

We conducted the pretest for Study 3 simultaneously with that of Study 2. To eliminate the potential interference caused by participants’ familiarity with the products, a virtual rice package based on authentic traditional production techniques was designed for Study 3. The pretest adopted a between-subjects design with a single factor and two levels (verbal vs. non-verbal). After viewing either the verbal or non-verbal heritage packaging images (as shown in Figure A3), participants were asked to evaluate the product packaging. The procedure was identical to the pretest in Study 2. Finally, participants reported their demographic information.

The independent samples t-test results showed that there was a significant difference in the scores between verbal heritage elements and non-verbal heritage elements in the experimental materials of Study 3. In terms of verbal perception scores, the verbal group scored higher (M_verbal_ = 5.92, SD = 1.04; M_non-verbal_ = 2.88, SD = 1.48, t(43) = 8.41, *p* < 0.001). In terms of non-verbal perception scores, the non-verbal group scored higher (M_verbal_ = 2.56, SD = 1.69; M_non-verbal_ = 5.80, SD = 1.23, t(48) = −7.78, *p* < 0.001). This indicates that the two types of heritage element representation style stimuli passed the test. Additionally, the results of participants’ ratings on packaging attractiveness showed no significant difference between the verbal and non-verbal groups (t(48) = −1.16, *p* = 0.25). The results of participants’ ratings on packaging informativeness also showed no significant difference between the verbal and non-verbal groups (t(48) = −1.83, *p* = 0.07). The results of participants’ ratings on packaging cultural authenticity also showed no significant difference between the verbal and non-verbal groups (t(48) = −1.84, *p* = 0.07). In sum-mary, participants were able to distinguish between the heritage element representation styles in the materials of Study 3, and alternative explanations based on packaging attractiveness, informativeness, and cultural authenticity were ruled out.

#### 3.4.2. Formal Experimental Design

A 2 (heritage cultural representation style: verbal vs. non-verbal) × 2 (purchase motivation: personal use vs. gift-giving) between-subjects experimental design was employed. Data were collected via the Credamo platform, with a total of 140 participants recruited, including 38 male participants (27.1%), 64 participants aged 31–40 years (45.7%), and 107 participants with a monthly consumption level of 982–1403 USD (76.4%). Prior to participation, each participant signed an online informed consent form.

#### 3.4.3. Method

Participants were randomly assigned to one of four groups. In the personal use scenario, participants were asked to imagine: “The rice stock at home is running low, and you plan to go to the supermarket for shopping. There are many people buying items at the supermarket, and you and your family have no particular preference for the taste of rice or any regular brand. The product you purchase mainly depends on your current impression, and you have sufficient funds for the purchase.” In the gift-giving scenario, participants were asked to imagine: “You plan to visit someone else’s house today and, for the purpose of gifting, you plan to purchase some rice gift boxes. The recipient has no preference for the taste of rice or any regular brand. The product you purchase mainly depends on your current impression, and you have sufficient funds for the purchase.” After reading the written materials and viewing the product packaging, participants in each group filled out the brand preference scale, perceived value scale, and manipulation check items, followed by reporting their demographic information. The scales for each variable used in this experiment are consistent with those in the previous studies.

#### 3.4.4. Results

*Manipulation check.* In terms of verbal perception scores, the verbal group scored higher (M_verbal_ = 4.42, SD = 1.35; M_non-verbal_ = 3.88, SD = 1.49, t(138) = 2.24, *p* = 0.027). In terms of non-verbal perception scores, the non-verbal group scored higher (M_verbal_ = 3.28, SD = 1.57; M_non-verbal_ = 5.04, SD = 1.34, t(135.9) = −7.14, *p* < 0.001). This indicates that the two types of heritage representation style stimuli passed the test.

*Moderation effect.* A two-way ANOVA was conducted with consumer brand preference as the dependent variable, and heritage element representation style and purchase motivation as the independent variables. The results showed that the interaction between the two factors is significant (F(1, 136) = 11.21, *p* = 0.001).

**Figure 7:**
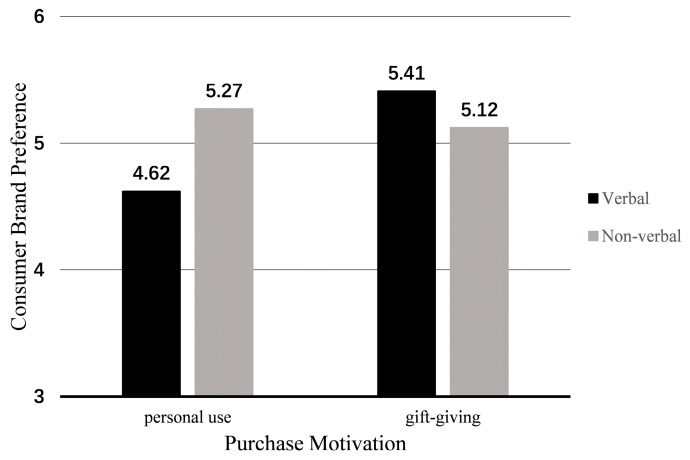
The moderating effect of purchase motivation in Study 3.

Further simple analysis showed that in the personal use scenario, participants in the non-verbal heritage element group had higher brand preference (M_verbal_ = 4.62, SD = 1.02; M_non-verbal_ = 5.27, SD = 0.63; F(1, 136) = 10.65, *p* = 0.001). In the gift-giving scenario, the verbal heritage element group had higher brand preference (M_verbal_ = 5.41, SD = 0.73; M_non-verbal_ = 5.12, SD = 0.86) (as shown in Figure 7), but the difference between the two groups was not significant (F(1, 136) = 2.13, *p* = 0.147). Thus, H4 was confirmed.

#### 3.4.5. Discussion

Whereas previous studies has primarily examined the asymmetric effects of different element styles based on consumers’ hemispheric lateralization and the spatial position of packaging elements [69,70], Study 3, based on a virtual food brand, examined the effect of purchase motivation on consumer decision-making in the context of heritage food product purchases. The results show that having a gift-giving motivation enhances the positive effect of the verbal heritage element representation style on consumer brand preference, while having a personal use motivation weakens the positive effect of the verbal heritage element representation style on consumer brand preference.

## 4. General Discussion

### 4.1. Conclusions

Unlike previous studies that mainly focus on the graphic features of heritage elements, this study, based on construal level theory, focuses on consumers’ value judgments regarding the heritage element representation style on food packaging. Through three experimental studies, it explores the preference differences, underlying mechanisms, and boundary conditions of consumer responses to different heritage cultural representation styles on food packaging. The following conclusions were drawn:

First, Study 1 demonstrated that food packaging containing heritage elements has a significant positive impact on consumer brand preference. Among these, the use of a verbal (vs. non-verbal) heritage element representation style more effectively enhances consumer brand preference. Additionally, it confirmed that perceived value mediates the effect of heritage element representation style on consumer brand preference.

Second, Study 2 again validated that in a tourism context, verbal heritage elements (vs. non-verbal heritage elements) more effectively increase consumer brand preference, and further proved that the mediating effect of perceived value in a tourism context still holds.

Third, Study 3 showed that purchase motivation moderates the main effect, with consumers purchasing for personal use showing a preference for the non-verbal heritage element representation style. In summary, all four hypotheses proposed in this study were supported.

### 4.2. Theoretical Contributions

This research enriches the literature on heritage food packaging. Existing research on heritage elements mainly focuses on commercialization and intellectual property, while studies on heritage product packaging include topics such as the impact of heritage packaging design symmetry [5], handmade scarcity [39], and geographical indications [71] on tourists’ emotions and behavior. These studies primarily focus on heritage tourism souvenirs, with relatively few examining heritage food packaging in the everyday consumer market, most of which focus on the integration of overall packaging design with local characteristic elements [72,73]. This research expands the application context of heritage food by examining consumers’ preferences for different heritage element representation styles in everyday purchasing behavior. The findings provide valuable insights for promoting the commercialization of heritage food beyond its place of origin, offering a meaningful extension to existing research. By analyzing consumers’ perceived psychological distance toward heritage elements and the core characteristics of verbal and visual cues, this research effectively shortens consumers’ emotional response time when engaging with heritage food, offering packaging design recommendations that can enhance the long-term market performance of such products.

Second, it enriches the relevant research on the impacts of packaging elements on consumers. Although previous research has addressed the moderating effects of involvement, time pressure, and individual characteristics [13,74], and have identified the synesthetic effects of non-verbal elements [75]. However, previous studies have rarely explored the impact of verbal/non-verbal packaging elements on consumers’ emotions and cognition from a cultural perspective. Grounded in construal level theory, this research verifies the close association between verbal packaging elements and the temporally distant nature of intangible cultural heritage. The findings provide empirical evidence for the moderating effects of verbal and non-verbal packaging elements within a cultural context.

Third, it verifies that different purchase motivation moderate consumer brand preference under the context of heritage element representation styles. Currently, most studies on heritage product marketing are based on the perspective of self-use as the purchase motivation, discussing the impacts of narrative expression, heritage labels, and a sense of awe on consumers’ perceptions [11,39,76]. Building upon this foundation, this research focuses on the distinct product cues that consumers attend to under different purchase motivations. It reveals a matching effect between the psychological distance shaped by diverse purchase purposes and the construal level of heritage elements in product packaging, offering a comprehensive explanatory framework for consumers’ purchase and experience intentions in the marketing of heritage products.

### 4.3. Managerial Implications

The current work also offers valuable practical implications for marketers and designers entrusted with developing innovative ideas for new heritage food designs. First, when designing food packaging, brands should select appropriate ways to display heritage elements, with a focus on highlighting the heritage cultural components. For instance, the heritage characteristics of the food or the heritage craftsmanship used can be prominently marked on the packaging to attract consumers’ attention and enhance their perceived value, leaving a positive impression of the brand. Regarding the use of element representation styles, priority should be given to verbal heritage elements that directly convey the heritage information of the food, facilitating effective information processing by consumers. If non-verbal heritage representation style elements are used, clear and concise graphic designs should be employed to communicate the beauty and craftsmanship of the heritage, ensuring the message is easily understood.

Second, strengthen perceived value awareness. Perceived value can mediate the impact of the heritage element representation style on consumer brand preference in food packaging. Relevant stakeholders should delve deeper into the cultural connotations of heritage products, employing narrative and storytelling transformation strategies, and effectively integrating them into food packaging. During the promotion phase, emphasis should be placed on promoting the heritage value of the food, telling the brand’s heritage story, and using heritage culture to convey brand emotions, which will help enhance consumers’ brand associations.

Third, establish a matching mechanism between usage purpose and product packaging. When designing heritage food packaging, companies should consider the product’s intended use and target audience in advance. They should apply appropriate heritage cultural representation styles on different functional packaging, ensuring that the usage-purpose-price-packaging heritage elements are aligned. This will enhance consumers’ information processing ability when encountering the product, thus driving sales. For example, on gift box packaging suitable for gifting, emphasis should be placed on using verbal heritage elements, while on regular packaging for personal use, unique non-verbal heritage elements should be showcased.

### 4.4. Limitations

Our findings also contain some limitations and present several avenues for future research. First, we classify representation styles into two categories: verbal and non-verbal. However, existing heritage elements often feature a “combination of graphics and text”. In future research, the impact of different proportions of graphics (vs. text) on product packaging or their different positions on packaging on consumer brand preference could be explored. For example, graphic elements on packaging can influence consumers’ perception of weight [77], while in the context of ICH, consumers hold a sense of awe toward ICH [76]. A question remains: Does this sense of awe affect the perceptions that consumers form through packaging elements? Second, this study mainly considers the moderating role of purchase motivation. Beyond this, some scholars have demonstrated that consumers’ knowledge level and time pressure can moderate the impact of verbal elements on consumers [12,74]. However, in the context of ICH, these factors may be interfered with by the cultural value of ICH, and whether their moderating roles remain effective requires further investigation. Third, all participants recruited in this research were from China. China is rich in heritage resources, which makes the impact of cultural identity more prominent among Chinese consumers. Nevertheless, ICH is regarded as a cultural heritage of the entire world and humanity, and its rationalized cross-border communication is expected to be achieved [8]. Therefore, future research can expand the scope of participants to explore the preferences of consumers from different national backgrounds regarding the representation styles of heritage elements in heritage food.

## Figures and Tables

**Table 1:** Items of each scale.

Variable	Scale	References
Consumer Brand Preference	When I need to purchase related products, this brand would be my first choice.	Jamal and Goode [63]
	Compared to other similar brands, I choose this brand not just because of the product itself.	
	I pay attention to information related to this brand and prefer maintaining a positive interactive relationship with it.	
	I am willing to recommend this brand’s products to family and friends.	
	I tend to repurchase this brand’s products to reduce associated decision-making costs.	
Brand Purchase Intention	I am likely to purchase products from this brand in the future.	McClure and Seock [64]
	I hope to purchase products from this brand in the future.	
	I plan to purchase products from this brand in the future.	
Perceived Value	You feel that purchasing this product will earn you more praise from those around you.	Sweeney and Soutar [65]
	You feel that purchasing this product will help you build a good social image.	
	You feel that purchasing this product will leave a good impression on others.	
	You feel that purchasing this product will help you gain social recognition.	
Processing Fluency	The information provided on the packaging is clear.	Septianto et al. [66]
	The information provided on the packaging is easy to understand.	
Emotional Arousal	Purchasing this product is exciting.	Güzel [67]
	Purchasing this product is thrilling.	
	Purchasing this product is enjoyable.	
	Purchasing this product is fun.	

## Data Availability

The original data presented in the study are openly available in FigShare at https://figshare.com/s/415b71b1714ca7f424da (accessed on 6 November 2025).
